# Quinine Flower

**Published:** 1882-10-20

**Authors:** 


QUININE FLOWER.—Dr. F H. Caldwell, of Sanford, Florida, has kindly sent us a specimen of the (*Sabbatia Ellrottii*) used in Florida for intermittent fever, which it is said to relieve in the same manner as quinine, producing the tinnitus aurium as does the sulphate of quinine. It is a small weed with a delicate yellowish flower. We thank the Doctor, and will deposit his specimen in the Botanical department of The Southern Medical College museum.

